# Integrative transcriptomic analysis reveals microglial metabolic-inflammatory crosstalk of HK2–HSPA5–TNF axis after intracerebral hemorrhage

**DOI:** 10.3389/fbinf.2025.1740715

**Published:** 2026-01-12

**Authors:** Yi Zhang, Yongqian Liu, Wei Meng, Xiaobo Yu, Xiaojun Xu

**Affiliations:** 1 Department of Radiology, The Second Affiliated Hospital, Zhejiang University School of Medicine, Hangzhou, China; 2 Key Laboratory of Precise Treatment and Clinical Translational Research of Neurological Diseases, Hangzhou, China; 3 Department of Radiology, The Second Affiliated Hospital of Jiaxing University, Jiaxing, China; 4 Department of Neurosurgery, The Second Affiliated Hospital, Zhejiang University School of Medicine, Hangzhou, China

**Keywords:** bioinformatics, intracerebral hemorrhage, metabolic reprogramming, microglia, neuroinflammation

## Abstract

**Background:**

Intracerebral hemorrhage (ICH) triggers secondary brain injury through neuroinflammation, yet the interplay between metabolic reprogramming and inflammatory responses remains poorly defined. This study investigated how glucose metabolism dysregulation contributes to neuroinflammatory pathogenesis following ICH.

**Methods:**

We integrated transcriptomic datasets from bulk RNA sequencing (human perihematomal tissue), single-cell RNA sequencing (mouse ICH model), and spatial transcriptomics (mouse time-series). Bioinformatic analyses included differential expression screening, single-cell weighted gene co-expression network analysis, pseudotemporal trajectory reconstruction, and cell-cell communication inference to identify key metabolic-inflammation regulators and their spatiotemporal dynamics.

**Results:**

Multi-omics convergence revealed hexokinase 2 (HK2), heat shock protein A5 (HSPA5), and tumor necrosis factor (TNF) as core regulators linking glucose metabolism to neuroinflammation. Single-cell analysis showed significant time-dependent regulation of HK2 in microglia, while spatial transcriptomics uncovered synchronized alterations of HK2, HSPA5, and TNF in perihematomal regions at day 7. Cell communication analysis highlighted enhanced microglia-to-neutrophil signaling via Tnf-Tnfrsf1b pairs, with TNF signaling identified as the most significantly upregulated pathway in ICH conditions.

**Conclusion:**

Our multi-omics approach reveals coordinated dysregulation of glucose metabolism and inflammatory genes following ICH, with time-dependent HK2 regulation in microglia and synchronized transcriptional changes at day 7 representing critical events in neuroinflammatory progression. The identified gene networks and cellular communication patterns provide new insights into the metabolic-immune interface in ICH, offering potential targets for future therapeutic strategies.

## 1 Introduction

Intracerebral hemorrhage (ICH) is a severe type of stroke with high rates of death and disability. While the initial bleeding causes direct damage, a major contributor to ongoing brain injury and poor recovery is the body’s inflammatory response that follows, known as neuroinflammation (Feigin et al., 2019; An et al., 2017). This involves complex interactions between brain cells like microglia and astrocytes, as well as immune cells entering from the bloodstream (Lan et al., 2017; Seiffge et al., 2024; Zhou et al., 2014).

Recent research across various neuropathological conditions—including metabolic diseases such as diabetes, as well as neurological disorders like Alzheimer’s and Parkinson’s disease—has increasingly demonstrated that dysregulation of energy metabolism, particularly glucose utilization, is closely linked to inflammatory processes (Alsbrook et al., 2023; Bahadar and Shah, 2021; Ovalı and Perçin, 2024). Changes in glucose metabolism pathways don't just happen alongside inflammation; they actively shape how severe and prolonged the inflammation becomes. For instance, metabolic shifts within immune and glial cells not only accompany inflammation but actively influence its intensity and duration (Bahadar and Shah, 2021; Gong et al., 2025; Han et al., 2021). Conversely, altered glycolytic flux in microglia can amplify pro-inflammatory signaling, while inflammation itself impairs key enzymes and glucose transporters, creating a vicious cycle of deterioration (Ma et al., 2022; Li Y. et al., 2024; Yao et al., 2025; D'Onofrio et al., 2021). However, despite evidence from these related fields, the specific mechanisms and regulatory genes governing the interaction between glucose metabolism and neuroinflammation following ICH remain poorly understood. Elucidating how these processes interact in ICH is crucial for identifying novel therapeutic strategies to mitigate secondary injury and improve neurological outcomes.

Technologies for studying gene activity (transcriptomics) have become vital tools. Bulk RNA sequencing (bulk RNA-seq) measures gene expression in whole tissue samples, giving an overall picture (Thind et al., 2021). Single-cell RNA sequencing (scRNA-seq) allows us to look at the gene activity of individual cells, revealing the specific roles of different cell types in the injured brain (Li and Wang, 2021). Spatial transcriptome RNA sequencing (stRNA-seq) takes this a step further by showing exactly where in the brain tissue (like near the bleed core or farther away) these gene expression changes occur (Piwecka et al., 2023). However, analyzing this complex data to specifically understand glucose metabolism’s role has been difficult. A common limitation in previous transcriptomic studies of ICH is their reliance on finding the most significantly changed genes (differentially expressed genes or DEGs). Genes related to glucose metabolism often show smaller, subtler changes in activity compared to genes directly involved in inflammation or immediate stress responses. Because these metabolic changes might not be the most dramatic statistically in standard analyses, they tend to receive less attention or can even be missed entirely, despite their fundamental biological importance (Wu et al., 2025; Tonyan et al., 2022; Yin et al., 2024).

Therefore, our study takes a different approach. We deliberately focus our bioinformatic analysis on genes known to be involved in glucose metabolism pathways. Using publicly available datasets generated from bulk RNA-seq, scRNA-seq, and stRNA-seq, we aim to identify even subtle changes in these glucose-related genes after ICH. By combining the strengths of these methods – the overall view from bulk data, the cell-type detail from scRNA-seq, and the location information from stRNA-seq – we set out to: 1) Pinpoint which glucose metabolism genes change in specific brain cells and their changes around the hematoma; 2) Investigate how these changes connect with known pathways of neuroinflammation and immune cells. We hypothesized that a coordinated dysregulation of glucose metabolism within specific neuroimmune cell populations drives the pathological progression of neuroinflammation following ICH. We hope to reveal novel links between energy metabolism failure and inflammation in the injured brain after hemorrhage. Understanding these specific connections may open up new therapeutic avenues for protecting the brain and reducing inflammation following ICH.

## 2 Methods

### 2.1 Multiple datasets and preprocessing

Transcriptomic data integration encompassed bulk RNA-seq (GSE24265: a microarray dataset from human beings with 4 perihematomal tissues and 7 contralateral ones (Rosell et al., 2011)), single-cell RNA-seq (GSE167593: whole brain tissues from 4 ICH and 4 control mice (Shi et al., 2021) at 14 days post-ICH), and spatial transcriptomics (STT0000047 in STOmics DB: whole brain tissues obtained at 3, 6, and 12 h and days 1, 3, 7, 14, and 28 post-ICH(23)).

Bulk data preprocessing involved the “sva” package in R language (version 4.1.0) to remove the batch effects (Leek et al., 2012). For scRNA-seq, gene counting was accomplished from the raw FASTQ data by “Cell Ranger” (version 7.0.1) software in Linux, while batch effects removal and clusters identification was achieved by Seurat package (version 4.2.1) (Hao et al., 2021). The Seurat pipeline implemented stringent quality control: cells with >200 detected genes and <99% maximum gene counts and <25% mitochondrial reads were retained, followed by normalization to mitigate technical variance. Batch correction employed canonical correlation analysis (CCA) integration, which preserves biological variation while removing dataset-specific artifacts in reduced dimension space. Processed spatiotemporal transcriptomic data at single-cell resolution was obtained from STMICH (https://db.cngb.org/stomics/stmich/).

### 2.2 Differentially expressed genes and functional annotation

Bulk differentially expressed genes (DEGs) analysis utilized limma’s empirical Bayes framework (Ritchie et al., 2015), modeling expression as a function of hemorrhagic or control tissues for batch effects through design matrix incorporation. Significance thresholds (|log_2_FC|>1, adj. p < 0.01) were determined via moderated t-statistics with Benjamini-Hochberg correction. To resolve the challenge of detecting subtle metabolic dysregulation, we implemented a sequential enrichment strategy. Initial Gene Ontology (GO) analysis of DEGs identified marginally enriched metabolic terms through clusterProfiler package in R language (Yu et al., 2012). We then utilized Gene Set Enrichment Analysis (GSEA) database to collect significantly enriched pathways and genes related to glucose metabolism (Supplementary Materials S2). These pathway-derived genes were intersected with our DEGs, yielding our final set of target metabolic genes. The biological coherence of these prioritized genes was validated through KEGG pathway enrichment analysis, confirming significant overrepresentation.

### 2.3 Single-cell clustering and annotation

Cellular heterogeneity resolution combined graph-based clustering with marker-driven annotation. The anchor-based integration algorithm (resolution = 0.09) partitioned cells in a shared nearest-neighbor graph constructed from 15 principal components by “IntegrateData” function in Seurat. Cell type annotation was initiated using the scCATCH package (version 3.2.2) (Shao et al., 2020), which implements tissue-specific marker database matching with statistical validation of cluster marker specificity. The “FindAllMarkers” function in Seurat was utilized to explore of gene expression differences across clusters, employing the Wilcoxon test to ascertain statistical significance (p.adj < 0.05) with default settings. This automated annotation was subsequently refined through manual curation using established cell-specific markers (e.g., Tmem119 for microglia and S100a9 for neutrophil (Zhang et al., 2019)).

### 2.4 Single-cell weighted gene co-expression network construction

Single-cell weighted gene co-expression dynamics were modeled using hdWGCNA’s metacell approach (Morabito et al., 2023). Gene selection was performed using a custom gene list with a minimum expression fraction threshold of 0.05 across cells. Metacells were generated via k-nearest neighbor aggregation (k = 30) within condition groups defined by both cell type and experimental group, with a maximum shared cell limit of 10 per metacell and cell type as the identity group. Signed network construction employed soft-thresholding power, determined by scale-free topology criterion with network type set to ‘signed’. Hierarchical clustering with dynamic tree cutting (minModuleSize = 50) identified modules, while module eigengene dissimilarity (mergeCutHeight = 0.2) guided merging. This analysis defines groups of co-expressed genes as “modules”. Module eigengenes were computed with group variation accounted for by experimental group, and intramodular connectivity (kME) was calculated to identify hub genes. Genes that do not associate strongly with any coherent cluster are assigned to a “gray” module, which serves as a benchmark against the “non-gray” modules (well-defined clusters of primary biological interest).

To elucidate functional interactions between co-expressed gene modules and prioritized metabolic regulators, we constructed a protein-protein interaction (PPI) network integrating two key elements: (1) hub genes identified from each hdWGCNA module (top 5 highest kME genes per module), and (2) our previously filtered glucose metabolism regulators. The PPI framework was built using the STRING database (version 12.0; confidence score >0.7; https://string-db.org/) with physical binding evidence requirements. Network topology analysis employed Cytoscape (version 3.10.3) using the CytoHubba plugin, which implemented maximal clique centrality to identify topologically critical nodes.

### 2.5 Microglia sub-clusters analysis

Microglia sub-clusters were resolved through iterative graph-based clustering at resolution 0.15. Transcriptionally distinct states of microglia (“Homeostatic_Microglia”, “Disease-Associated Microglia”, “M1_Cell”, “M2_Cell”, “Proliferating_Cell”, and “Lipid-Associated Microglia”) were annotated according to previous references (Zhang et al., 2024; Zhu et al., 2024). Pseudotemporal trajectories were reconstructed using Monocle3’s manifold learning framework (version 1.3.7) (Trapnell et al., 2014). The principal graph was initialized through reversed graph embedding as Equation 1:

$$\min_{f,Q}\sum_{i}{\left\|x_{i}-f\left(q_{i}\right)\right\|}^{2}+\lambda {\left\|\nabla f\right\|}^{2}$$ (1)

where $x_{i}$ represents gene expression vectors and $q_{i}$ denotes manifold coordinates (*λ* = 0.01). Rooting was biologically anchored to homeostatic cells (pseudotime *τ* = 0). Gene expression kinetics along trajectories were modeled via generalized additive models with thin-plate splines as Equation 2:

$$g\left(E_{g}\right)=\beta_{0}+f_{g}\left(\tau \right)+\epsilon$$ (2)

where $f_{g}$ represents the spline function (k = 10 basis functions) for target genes, incorporating group (ICH vs. control) as covariates to identify condition-specific changes. Temporal trends were visualized through custom LOESS-smoothed (span = 0.75) expression plots with 95% CIs.

For visualization and analysis of gene expression dynamics along pseudotime (Figures 4C–E), the average expression level was calculated solely based on cells with detectable expression (non-zero counts) for the respective gene. This approach focuses on the expressing cell population and provides a clearer representation of transcriptional dynamics without dilution by non-expressing cells (Wu et al., 2025; Vallejos et al., 2017). This analytical strategy was particularly important for capturing the subtle expression patterns of glucose metabolism-related genes, which typically exhibit low-abundance signals that would otherwise be obscured in bulk cell analyses.

### 2.6 Spatial transcriptomics analysis

Spatial transcriptomics analysis leveraged preprocessed Visium data from lesional (hemorrhage-affected) and contralateral hemispheres across nine timepoints (Naive to D28). Microglia-specific spatial spots were identified through reference annotation. For each target gene, temporal expression proportion were normalized to naive-state baselines as Equation 3:

$$E_{norm}=\frac{E_{t}}{E_{naive}}$$ (3)

where $E_{t}$ is the expression proportion at timepoint t and $E_{naive}$ is the average expression percentage in naive animals. Temporal dynamics were quantified through two complementary metrics: expression abundance (normalized proportion of expressing microglia spots) and expression magnitude (mean expression level across expressing spots).

### 2.7 Cell-cell communication analysis

Cell-cell communication analysis were accomplished using CellChat (version 2.1.2) (Jin et al., 2025). The analysis was conducted separately for control and ICH groups using the mouse database (CellChatDB.mouse). Given the absence of established interactions for key metabolic genes in standard databases, we augmented CellChatDB with experimentally validated ligand-receptor pair of our target genes. We custom-designed the Hspa5-related pathway based on literature references, incorporating known interactions involving Hspa5 (a key endoplasmic reticulum chaperone implicated in stress response and neuroinflammation) to ensure comprehensive coverage of metabolic-immune crosstalk in neurological contexts. These interactions were selected from STRING (confidence>0.7) and literature evidence of physical binding in neurological contexts. Key computational parameters included: communication probability calculation using a truncated mean model with trim = 0.25 to reduce outlier effects; minimum cell group size threshold of 10 cells (min.cells = 10) for interaction filtering; and non-protein interaction allowance (non_protein = TRUE) to capture comprehensive interaction types. Communication probabilities mean model as Equation 4:

$$P_{ij}=\sum_{k}w_{k}\cdot \frac{1}{m}\sum_{r=1}^{m}T_{r}\left(L_{k},R_{k}\right)$$ (4)

where $T_{r}$ trims 25% extremes of ligand/receptor expressions, $w_{k}$ weights interactions by frequency, and m denotes metacells. Differential pathway engagement (ICH vs. control) was assessed through 10,000-label permutations, with FDR correction for ligand-receptor family dependencies. Network centrality metrics were computed using netAnalysis_computeCentrality () to identify topologically critical nodes in the neuroinflammatory-metabolic crosstalk network.

### 2.8 Animal models of subarachnoid hemorrhage and scRNA

All animal experiments were conducted in accordance with the National Institutes of Health Guide for the Care and Use of Laboratory Animals. The protocols were reviewed and approved by the Institutional Animal Care and Use Committee of Zhejiang University. Adult male C57BL/6 mice (aged 8–10 weeks, weighing 22–25 g) were purchased from SLAC Laboratory Animal Co., Ltd. (Shanghai, China). Subarachnoid hemorrhage (SAH) was induced via endovascular perforation following established protocols (Fujimoto et al., 2016).

For scRNA-seq preparation, fresh brain tissues (1 day after SAH) were rapidly dissected and transferred into cold Hibernate A solution (BrainBits, LLC) to maintain cellular viability. Tissue dissociation was performed. The digested tissue was mechanically triturated 20 times using a 5 mL serological pipette and filtered. After centrifugation, the pellet was resuspended and centrifuged to remove myelin debris and enrich viable cells. Erythrocytes were lysed using ACK lysing buffer. Single-cell suspensions were loaded onto a Chromium Single Cell B Chip (10x Genomics) targeting a recovery of 8,000–10,000 cells per sample, and scRNA-seq libraries were constructed using the Chromium Single Cell 3′ Reagent Kit v3.1 according to the manufacturer’s protocol. All subsequent bioinformatic analyses, including quality control, normalization, clustering, and differential expression, were performed as described in the Methods section above.

Note: The SAH model data described in this section were generated exclusively to provide supplementary context (Supplementary Figure S3) for the discussion of Hk2 dynamics across hemorrhagic stroke models.

### 2.9 Statistical validation and reproducibility

All inferences incorporated rigorous multiplicity control. False discovery rates were estimated via Benjamini-Hochberg for independent hypotheses and Benjamini-Yekutieli for dependent tests. Effect sizes for differential interactions were reported as Cohen’s d with 95% confidence intervals from 10,000 bootstrap samples. Reproducibility was guaranteed through Docker containerization by tidyverse (version 2.0.0) (Kandel et al., 2011), with computational environments frozen at analysis runtime. Parameter configurations were version-controlled via Git, and all random processes were seeded (seed = 349) for deterministic execution.

## 3 Results

### 3.1 Bulk RNA-seq analysis focused on dysregulated glucose metabolism genes

To establish whether glucose metabolic pathways are transcriptionally altered following ICH, we first analyzed bulk RNA-seq dataset (GSE24265) comprising human perihematomal tissues and contralateral controls. Using a targeted enrichment strategy focused on glucose metabolism, we performed standard differential expression analysis and enrichment analysis, followed by intersection with gene sets and pathway lists from public GSEA resources (Supplementary Figures S1A–D).

As anticipated, differential expression analysis revealed a limited number of genes with modest changes: 11 were upregulated (Cxcr4, Tgfbi, Isg20, Tktl1, Slc2a3, Hspa5, Hk2, P4ha1, Slc2a1, Stc2, B4galt1) and two were downregulated (Sox9, Foxk1), as shown in the volcano plot with relatively low log_2_FC and -log_10_ (p-value) values (Supplementary Figure S1B). These genes were selected for subsequent analyses. Gene Ontology (GO) analysis highlighted processes primarily associated with immediate stress responses and inflammatory pathways, consistent with previously reported outcomes. Only two biological processes were directly related to glucose metabolism: “response to nutrient levels” and “cellular response to glucose starvation” (Supplementary Figure S1D). Furthermore, KEGG pathway enrichment analysis affirmed the biological coherence of this gene set and supported its central role in metabolic processes (Supplementary Figure S1C), suggesting a potential rewiring of central carbon metabolism in the human brain following ICH. These results confirmed subtle but coherent dysregulation of glucose metabolic genes in human ICH tissues, justifying a higher-resolution investigation into their cell-type-specific expression.

### 3.2 ICH induces inflammation activation and alters glucose metabolism

**FIGURE 1:**
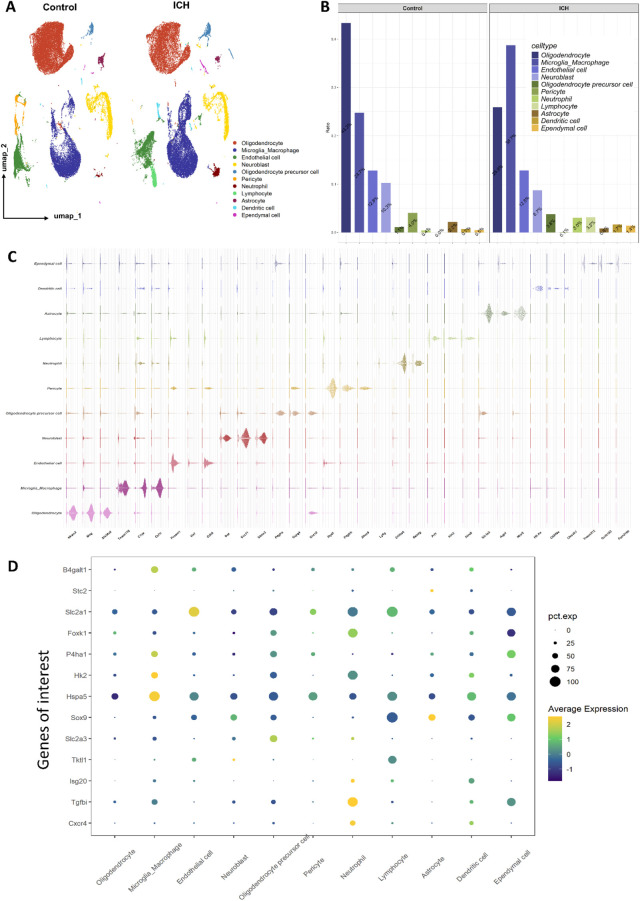
Cellular composition changes and gene expression alterations following ICH. **(A)** UMAP visualization shows the distribution of 13 major cell types between control and ICH conditions. **(B)** Bar plot compares proportional changes in cell populations, with microglia/macrophages and neutrophils significantly increased in ICH group. **(C)** Violin plots displays gene markers of cell clusters. **(D)** Dot plot shows expression patterns of genes of interest across cell types, with point size indicating the percentage of cells expressing the gene (pct.exp) and color representing average expression level.

Single-cell RNA sequencing analysis revealed substantial alterations in the cellular composition following ICH. The UMAP (uniform manifold approximation and projection) visualization demonstrated distinct clustering patterns between control and ICH conditions, with microglia/macrophages showing expanded distribution in ICH samples alongside the emergence of neutrophil populations (Figure 1A). Quantitative analysis revealed significant changes in cell type proportions, with microglia/macrophages increasing from 24.7% in controls to 38.7% in ICH conditions, while neutrophils increased dramatically from 0.4% to 3.0% (Figure 1B). These shifts indicate substantial immune cell infiltration and microglial population changes following hemorrhagic injury. Cell type annotation was confirmed through violin plots displaying established marker genes, including Tmem119 for microglia, Gfap for astrocytes, and S100a9 for neutrophils, which showed distinct expression patterns validating the classification accuracy (Figure 1C).

Having established the overall inflammatory cellular landscape, we next asked whether our prioritized glucose metabolism genes exhibited cell-type-specific expression patterns that could explain their potential role. Analysis of selected genes of interest across cell types revealed cell-specific expression patterns particularly relevant to glucose metabolism and inflammatory responses (Figure 1D). The glucose metabolism genes Hk2 and Hspa5 showed preferential expression in microglia/macrophages, while the genes Cxcr4 and Tgfbi exhibited elevated expression in neutrophils. These findings demonstrate that ICH induces not only population-level changes in microglial and neutrophil abundance but also specific upregulation of key genes involved in metabolic and inflammatory responses within these critical immune cell types.

### 3.3 Single-cell WGCNA reveals key modules associated with neuroinflammation and metabolic reprogramming after ICH

**FIGURE 2:**
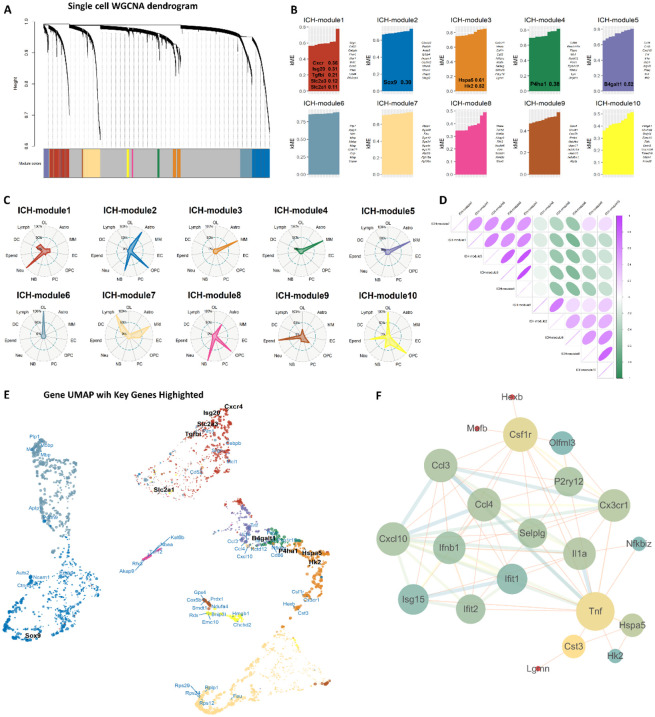
Single-cell WGCNA reveals functional modules and their cellular associations. **(A)** Hierarchical clustering dendrogram of co-expression modules. **(B)** Bar plot displaying module membership (kME values) of hub genes and selected genes across all modules. **(C)** Radar plots illustrating cell type enrichment proportion for each co-expression module. Cell type abbreviations: Oligodendrocyte (OL), Microglia/Macrophage (MM), Endothelial cell (EC), Neuroblast (NB), Oligodendrocyte precursor cell (OPC), Pericyte (PC), Neutrophil (Neu), Lymphocyte (Lymph), Astrocyte (Astro), Dendritic cell (DC), Ependymal cell (Epend). Radial axis represents normalized enrichment proportion (0%–100%). **(D)** Module correlation heatmap showing Pearson correlation coefficients between module eigengenes. Purple indicates positive correlation, green indicates negative correlation. **(E)** UMAP visualization of genes across co-expression modules, with hub genes and selected target genes highlighted. **(F)** Protein-protein interaction network of hub genes and selected genes. Key genes Tnf, Hspa5, and Hk2 are prominently showed, illustrating their potential interconnected relationships within the network.

To move beyond individual gene expression and uncover coordinated transcriptional programs that might link metabolic and inflammatory processes, we performed WGCNA. This approach identifies modules of co-expressed genes, revealing potential functional relationships that are not apparent in differential expression analysis alone. Hierarchical clustering identified 10 distinct co-expression modules (designated ICH-module1 to ICH-module10), each represented by unique colors and comprising genes with highly correlated expression patterns across cell types (Figure 2A). Module assignment revealed specific functional specialization, with selected genes of interest showing particularly high module membership (kME) values (Figure 2B). The kME metric, representing intramodular connectivity, quantifies how well each gene’s expression correlates with the module eigengene, with values approaching ±1 indicating strong positive or negative association. Comparative analysis between ICH and control conditions revealed significant differential module eigengene expression (Supplementary Figure S2A,B), with modules 1 and 4 showing marked upregulation in ICH groups. Functional enrichment analysis of module genes (Supplementary Figures S2C–E) demonstrated significant associations with key biological processes, providing mechanistic insights into the coordinated transcriptional reprogramming following ICH.

Notably, several modules exhibited characteristics associated with neuroinflammatory processes and, in part, metabolic functions. Module 3, for instance, contained both the endoplasmic reticulum stress regulator Hspa5 (kME = 0.61) and the glucose metabolism gene Hk2 (kME = 0.52), suggesting a possible interplay between metabolic reprogramming and cellular stress response within inflammatory cells. Module 1 was enriched for inflammatory response genes (e.g., Cebpb and Cd44), indicating its potential role in acute neuroinflammation. Radar plot analysis demonstrated cell type-specific contributions to module composition, revealing that microglia/macrophages showed predominant involvement in modules 3, 4, 5, and 7, while neutrophils contributed significantly to module 1 (Figure 2C). Module correlation analysis further revealed both positive and negative relationships among modules (Figure 2D). The strong positive correlation observed between module 1 (enriched in inflammatory genes) and modules 3–5 (which contain several metabolism-associated genes) may indicate coordinated regulation between neuroinflammatory responses and certain metabolic pathways, though further functional validation is warranted to establish direct mechanistic links.

UMAP visualization of module genes highlighted the spatial organization of co-expression relationships, with hub genes and selected target genes forming distinct clusters within the topological space (Figure 2E). Notably, Hspa5 and Hk2 occupied adjacent positions within the module 3 cluster, indicating their strong relationship. The spatial proximity between these key genes suggests potential functional interactions in the cellular response to hemorrhagic injury. Protein-protein interaction analysis further confirmed biologically relevant networks among hub genes (Figure 2F), with glucose metabolism genes forming interconnected subnetworks with inflammatory mediators. Crucially, Tnf, Hspa5, and Hk2 appeared as central nodes within their respective modules and showed direct protein-protein interactions, forming a triangular network that bridges inflammatory signaling (Tnf), endoplasmic reticulum stress response (Hspa5), and glucose metabolic processes (Hk2). This interconnected relationship suggests a molecular framework through which neuroinflammatory signals may regulate metabolic adaptation in glial cells following ICH. To validate this predicted interplay and investigate its cellular context, we next focused on microglial subpopulations.

### 3.4 Analysis of co-enrichment relationships and microglial subpopulation dynamics after ICH

**FIGURE 3:**
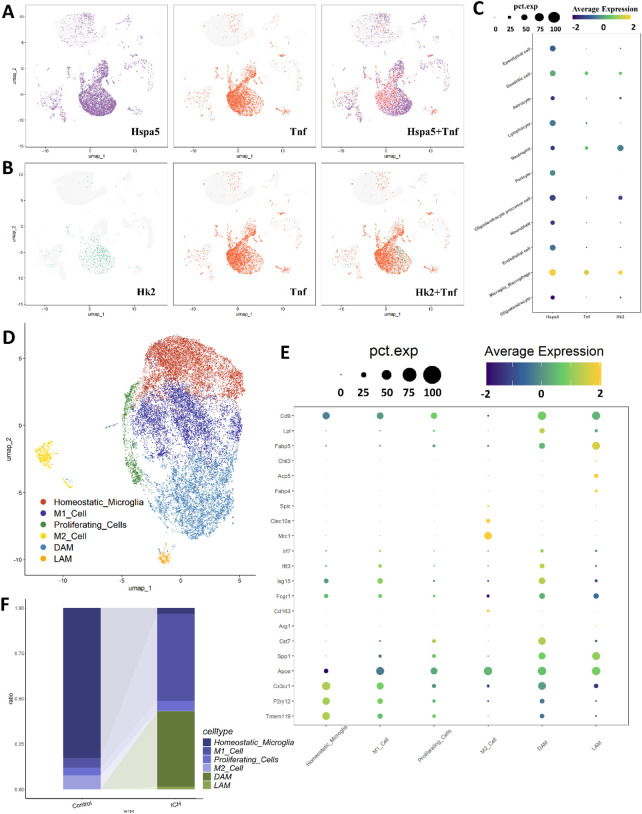
Co-enrichment relationships and microglial subpopulation after ICH. **(A,B)** The UMAP plots show co-enrichment relationships between **(A)** Hspa5 and Tnf, and **(B)** Hk2 and Tnf across microglial subpopulations. **(C)** The bubble plot illustrated the co-enrichment patterns of Hspa5, Tnf, and Hk2 in microglia. **(D)** UMAP visualization of microglia/macrophage clusters, showing six transcriptionally distinct subpopulations: homeostatic microglia, disease-associated microglia (DAM), M1-like polarized cells (M1_cell), M2-like polarized cells (M1_cell), proliferating microglia, and lipid-associated microglia (LAM). **(E)** Dot plot displaying marker gene expression for each microglial subpopulation. Dot size represents the percentage of cells expressing the gene (pct.exp), and color intensity indicates average expression level. **(F)** Stacked bar chart comparing proportional changes of microglial subpopulations between Control and ICH conditions.

Given the central role of microglia and the prominent placement of our target genes in microglia-enriched WGCNA modules, we sought to investigate their dynamics at a higher resolution within microglial subpopulations. We specifically investigated the co-enrichment relationships among Hspa5, Tnf, and Hk2 within microglial subpopulations. The UMAP plot revealed spatially significant correlations between Hspa5-Tnf and Hk2-Tnf expression pairs across microglial cells (Figures 3A,B). The bubble plot directly illustrated the co-enrichment patterns of Hspa5, Tnf, and Hk2 across all major cell types, particularly in microglia (Figure 3C). To further characterize the cellular contexts underlying these coordinated expression patterns, we performed high-resolution clustering of microglia/macrophage populations, identifying six transcriptionally distinct subpopulations (Figure 3D). UMAP visualization revealed clear separation among homeostatic microglia, disease-associated microglia (DAM), M1-like polarized cells (M1 cell), M2-like polarized cells (M2 cell), proliferating microglia, and lipid-associated microglia (LAM). Key markers of these microglial subpopulations were showed in Figure 3E according to reference publications. Figure 3F illustrates the shifts in cellular composition following ICH. A marked reduction can be observed in homeostatic microglia, accompanied by substantial expansions of DAM, M1-like, and LAM subpopulations, indicating widespread microglial activation and phenotypic transformation post-ICH. These alterations suggest a dynamic reprogramming of microglial states in response to hemorrhagic injury.

**FIGURE 4:**
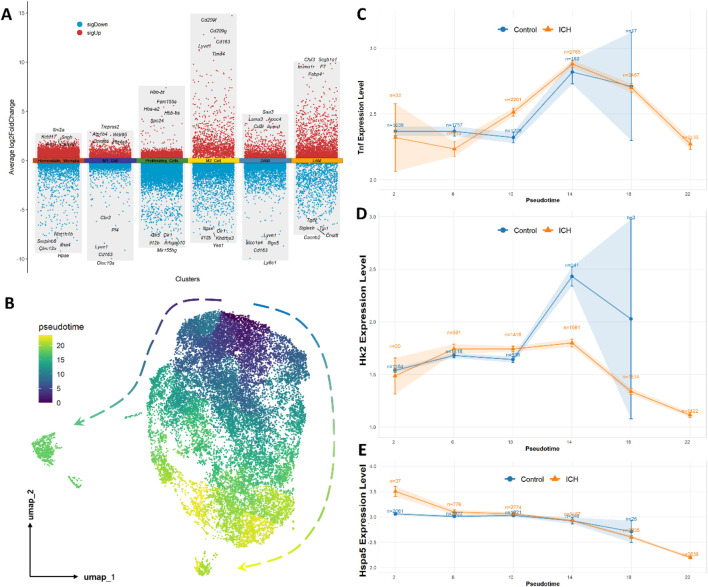
Pseudotemporal dynamics of gene expression and cellular distribution in microglial subpopulations. **(A)** Volcano plot displaying differentially expressed genes across microglial clusters. Red dots denote significantly upregulated genes, while blue dots indicate downregulated genes. **(B)** Reconstructed pseudotemporal trajectory suggesting two major differentiation pathways originating from homeostatic microglia. **(C–E)** Dynamics of Tnf, Hk2, and Hspa5 expression along pseudotime in Control (blue) and ICH (orange) groups. Expression levels were calculated based on cells with detectable counts (non-zero expression) to minimize dilution effects from non-expressing cells. Curves represent mean expression values, and shaded areas indicate 95% confidence intervals. Corresponding figures reflect the number of responsive cells for each gene at key pseudotime points.

Transcriptional profiling of microglial subpopulations revealed distinct expression patterns through volcano plot analysis (Figure 4A). Not surprisingly, metabolic genes like Hk2 were not seen in the top5 genes. Then, pseudotemporal trajectory analysis reconstructed two major differentiation pathways originating from homeostatic microglia. The analysis suggested two major trajectories: a primary inflammatory pathway progressed through homeostatic to M1-like polarization and DAM/LAM, while a secondary reparative pathway followed homeostatic to proliferating and M2-like differentiation (Figure 4B). These cellular transitions appear consistent with the state conversions observed in Figure 3, indicating possible reprogramming routes after ICH, with Pathway 1 showing substantial expansion under hemorrhagic conditions. However, as pseudotime analysis is computational and inferential, these pathways should be interpreted as hypothetical models rather than definitive biological processes.

Time-resolved analysis of gene expression and associated cellular distributions along pseudotime revealed distinct patterns between ICH and control groups (Figures 4C–E). In the Tnf-associated trajectory (Figure 4C), ICH samples exhibited markedly elevated expression levels that peaked at pseudotime point 14 (mainly M1-like cells), constituting 27.4% of total microglia, compared to only 3.97% in controls at the same timepoint. The Hk2-associated pathway (Figure 4D) demonstrated a characteristic rise-and-fall pattern in ICH conditions, with expression levels reaching maximum at pseudotime 14 (24.9% of responsive cells) before declining, contrasting with the stable low expression in controls (3.85% of responsive cells). Hspa5-associated dynamics (Figure 4E) showed a consistent decline in expression levels throughout the pseudotemporal trajectory in ICH conditions, with responsive cell proportions progressively increasing from 6.35% to 21.6% between pseudotime 6–18, while control samples maintained relatively stable expression (2.75–3.05). It is important to note that these pseudotime-based patterns may not directly reflect real-time biological dynamics which serves to generate hypotheses for further validation.

Notably, both Tnf and Hk2 exhibited similar expression patterns characterized by initial increase followed by subsequent decrease, which could suggest potential coordinated regulation during microglial activation. In contrast, Hspa5 demonstrated an opposing trend with persistent downregulation, possibly indicating a negative relationship with the inflammatory-metabolic activation represented by Tnf and Hk2. These contrasting dynamics may imply that Tnf and Hk2 participate in a coordinated manner in the microglial response to hemorrhagic injury, while Hspa5 appears to be inversely regulated, potentially representing counteracting pathways in the metabolic-inflammatory network activated following ICH.

### 3.5 Spatial transcriptomic analysis of selected genes through temporal lines

**FIGURE 5:**
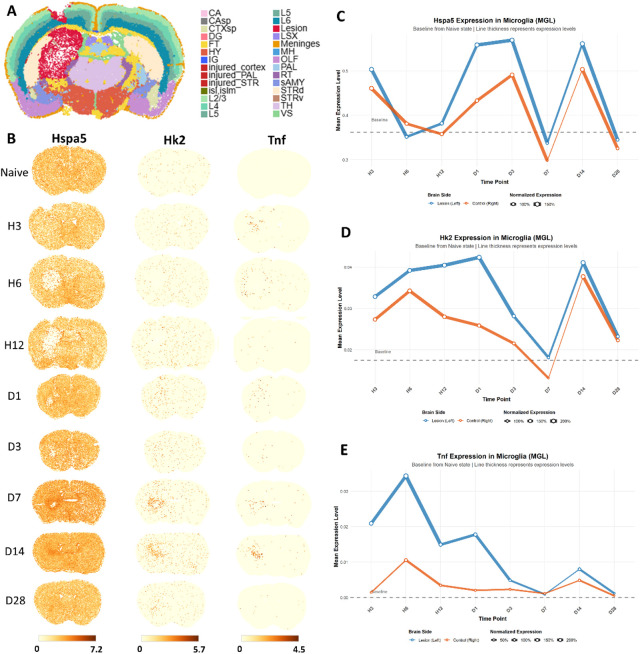
Spatiotemporal transcriptomic dynamics of selected genes across true timepoints. **(A)** Anatomical illustration of the autologous blood injection-induced ICH model in mice. Abbreviations denote: CA, Ammon’s horn; CAsp, field Ammon’s horn, pyramidal layer; CTXsp, cortical subplate; DG, dentate gyrus; FT, fiber tracts; HY, hypothalamus; injured_cortex, injured cortex region; injured_PAL, injured pallidum region; injured_STR, injured striatum region; isl, islands of Calleja; islm, major island of Calleja; L2/3, cortical layer 2 and cortical layer 3; L4, cortical layer 4; L5, cortical layer 5; L6, cortical layer 6; Lesion, Lesion region; LSX, lateral septal complex; MH, medial habenula; OLF, olfactory areas; PAL, pallidum; RT, reticular nucleus of the thalamus; sAMY, striatum-like amygdala nuclei; STRd, striatum dorsal region; STRv, striatum ventral region; TH, thalamus; VS, ventricular systems. **(B)** Spatial expression dot plot displaying spatial expression patterns of Hspa5, Hk2, and Tnf across multiple timepoints (H: hours; **(D)** days post-ICH) compared to the naive state. **(C–E)** Line plots showing temporal expression dynamics in microglia for Hspa5, Hk2, and Tnf in both lesion (left, orange) and control (right, blue) hemispheres. Y-axis represents mean expression level calculated from cells with detectable expression. Line thickness corresponds to the proportion of expressing cells (normalized to naive baseline).

To validate the pseudotemporal dynamics we observed and map them to true biological time and anatomical space, we performed spatial transcriptomic analysis in mouse models of ICH. Figure 5A illustrates the anatomical regions analyzed, with colored areas indicating distinct brain areas affected by the autologous blood injection (more detailed tissue section images are available in the original publications (Xiang et al., 2025)). Spatial expression analysis (Figure 5B) revealed temporally regulated patterns showing significantly elevated expression of Hspa5, Hk2, and Tnf surrounding the hematoma region (left hemisphere) compared to the contralateral side (right hemisphere) across several timepoints. Particularly, Hk2 and Tnf demonstrated the most pronounced spatial enrichment around the hemorrhage site, with consistent high expression patterns observed across consecutive timepoints, indicating coordinated spatial regulation of these genes in response to hemorrhagic injury.

Temporal dynamics analysis in microglia showed distinct expression patterns for each gene (Figures 5C–E). Hspa5 expression (Figure 5C) demonstrated sustained upregulation in the lesion hemisphere (blue line) throughout the time course, maintaining 1.2–1.5-fold higher expression compared to the control hemisphere (orange line) from D1 to D28. Hk2 expression (Figure 5D) exhibited a biphasic response, with initial activation at early timepoints (H3-H12) followed by progressive suppression from D1 onward, showing particularly strong expression in the lesion hemisphere. Tnf expression (Figure 5E) displayed an acute inflammatory response, peaking at H6-D1 in the lesion hemisphere with approximately 3-fold higher expression compared to controls, followed by gradual resolution by D7.

At D7 post-ICH, all three genes exhibited a remarkable simultaneous reduction in expression. Both the hematoma-surrounding region and contralateral hemisphere showed substantially diminished signals for Hspa5, Hk2, and Tnf compared to earlier timepoints, with expression levels dropping to or below naive baseline values. This transient global suppression may suggest a potential synchronized regulatory mechanism or cellular state transition occurring specifically at this timepoint in the ICH pathological progression.

### 3.6 Cell-cell communication analysis reveals altered inflammatory signaling networks after ICH

To investigate how the observed gene expression translate into altered cellular crosstalk, we performed a comprehensive cell-cell communication analysis focusing on microglia/macrophages and neutrophils. This analysis aimed to determine whether the metabolic and inflammatory genes identified in our previous results (particularly Hspa5, Hk2, and Tnf) participate in specific intercellular signaling pathways that are modified following ICH.

**FIGURE 6:**
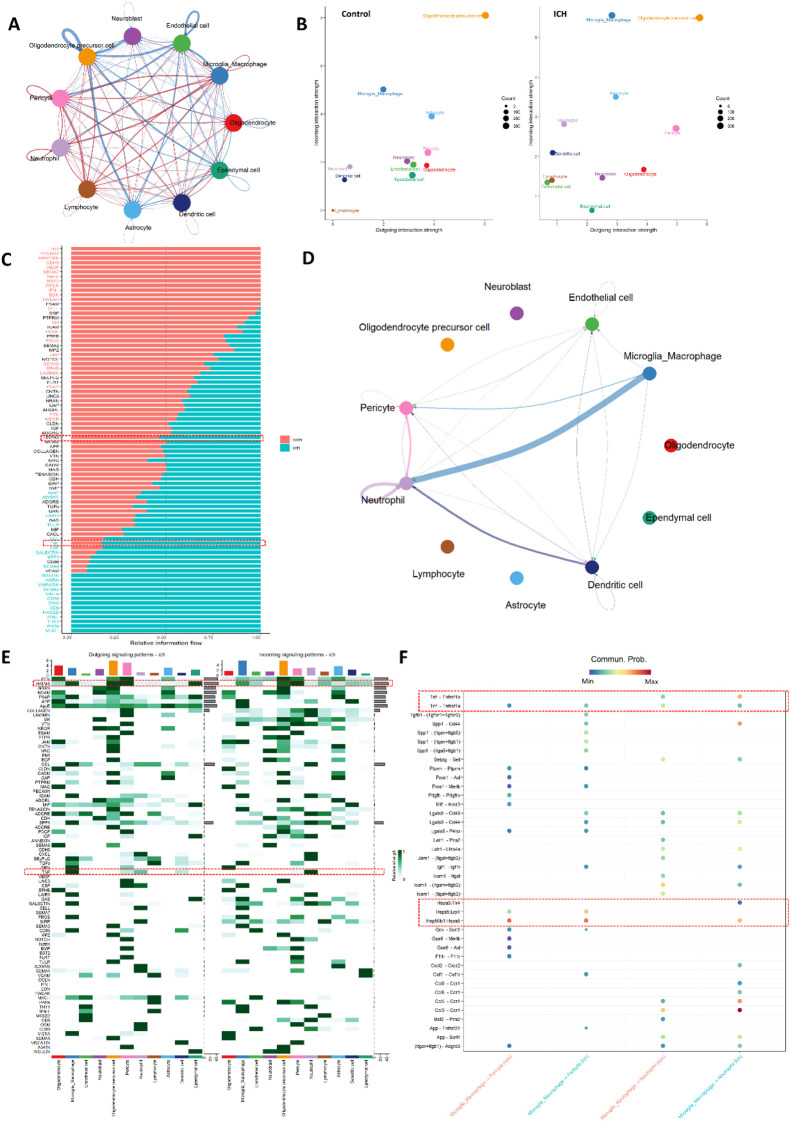
Cell-cell communication network remodeling after ICH. **(A)** Global communication network showing significantly enhanced signaling strength in ICH (red edges) versus control (blue). **(B)** Scatter plot shows alterations of outgoing and incoming communication strength between two conditions. **(C)** Stacked bar plot illustrates pathway-specific comparison in two groups. **(D)** Network visualization illustrating TNF signaling with paracrine (microglia→neutrophils) and autocrine (neutrophil self-communication) interactions. **(E)** Cellular communication patterns showing outgoing (left) and incoming (right) signaling for specified genes across cell types. Color intensity indicates communication probability. **(F)** Altered ligand-receptor pairs between microglia/macrophages and their communication partners. Color indicates interaction probability.

Global communication analysis revealed significantly enhanced signaling strength in the ICH group compared to controls (Figure 6A), with red edges indicating interactions that were predominantly elevated in ICH conditions. This was particularly evident in the communication involving microglia/macrophages and neutrophils, which showed the most substantial increases in signaling activity. Directional signaling analysis (Figure 6B) demonstrated distinct reorganization of signaling networks after hemorrhagic injury, with microglia/macrophages exhibiting increased outgoing (from 2.0 to 3.0) and incoming (from 5.0 to 5.5) weighted communication strength. Neutrophils showed even more pronounced changes, with incoming communication increasing from 1.8 to 2.8 and outgoing communication rising from 0.7 to 1.2, indicating their enhanced role in both receiving and sending signals in the ICH environment.

Pathway-specific comparison between groups (Figure 6C) identified TNF signaling as one significant upregulated pathway in ICH conditions, while Hspa5-and Hk2-associated pathways showed no significant intergroup differences. Detailed mapping of TNF signaling networks (Figure 6D) revealed two dominant communication modes in ICH: paracrine signaling from microglia/macrophages to neutrophils accompanied by autocrine signaling within neutrophils. These patterns demonstrate how TNF-mediated communication is amplified through both intercellular crosstalk and intracellular reinforcement in the inflammatory microenvironment post-ICH.

Cellular component analysis (Figure 6E) revealed cell-type-specific communication patterns for the highlighted genes. While Hspa5-associated signaling showed no significant differences between cell types, TNF signaling exhibited particularly strong outgoing communication from microglia/macrophages and incoming communication to neutrophils in ICH conditions, indicating a specific microglia/macrophages-to-neutrophil signaling axis. Finally, ligand-receptor pair analysis (Figure 6F) identified the most significantly altered specific interactions between microglia/macrophages and their major communication partners (neutrophils and pericytes) in ICH versus control conditions. These pairs included two TNF-related interactions that were substantially strengthened in ICH group, in which Tnf-Tnfrsf1b was the most significantly altered interaction in microglia/macrophage-to-neutrophil communication. While HSPA5-related pairs (e.g., HSPA5-LRP1) were detectable, from previous analysis they may show minimal condition-specific changes, suggesting baseline biological functions rather than ICH-specific roles. These findings mechanistically explain how TNF-mediated signaling drives inflammatory amplification through specific molecular cascades post-hemorrhage.

Together, these analyses demonstrate that TNF-mediated signaling undergoes the most substantial reorganization after ICH, particularly through enhanced microglia-to-neutrophil communication. This positions TNF as the primary intercellular messenger in the metabolic-inflammatory network, while Hspa5 and Hk2 appear to function more as intracellular regulators of cell state.

## 4 Discussion

Cerebrovascular accident triggers a complex neuroinflammatory response intertwined with profound metabolic alterations, yet the interplay between these processes remains poorly understood. Glucose metabolic pathways, being fundamental cellular functions, often exhibit subtler changes compared to dramatic inflammatory shifts, causing them to be frequently overlooked in conventional differential expression analyses focused on the most statistically significant changes (Lei et al., 2025). This study was specifically designed to overcome this limitation by employing a targeted approach to investigate how glucose metabolism and neuroinflammatory signaling interact at cellular and molecular levels following ICH.

Bulk tissue analysis confirmed that while glucose metabolism pathways showed only modest changes in overall expression, they demonstrated consistent and biologically relevant alterations across analytical platforms. These findings align with emerging concepts that metabolic reprogramming represents a fundamental component of neuroinflammation rather than a peripheral phenomenon (Li Y. et al., 2024; Thieren et al., 2025), while previous studies have established that glycolytic enzymes support immune cell activation and cytokine production (Namgaladze and Brüne, 2023). The concurrent expansion of DAM, M1-like cells, and LAM subsets suggests parallel activation of inflammatory, phagocytic, and lipid metabolic pathways in response to hemorrhagic injury. Notably, through single-cell WGCNA and subsequent PPI analysis, we identified three key regulators that appear to form a coordinated network: Tnf, Hk2, and Hspa5. These genes exhibited interconnected expression patterns and protein interactions suggesting functional relationships.

Previous research has demonstrated that TNF is a master regulator of neuroinflammation following ICH, primarily released by activated M1 microglia and macrophages. TNF amplifies the inflammatory cascade by promoting further microglial activation and recruiting peripheral leukocytes, while also contributing to excitotoxic neuronal death. Preclinical studies demonstrate that TNF inhibition attenuates edema and improves neurological outcomes (Tschoe et al., 2020; Jia et al., 2024). In the context of metabolic regulation, HK2 plays a divergent role under different neuropathological conditions. In ischemic stroke, HK2 influences microglial metabolic reprogramming and inflammatory modulation: dexmedetomidine (DEX) pretreatment upregulates HK2, enhancing glycolytic flux and oxidative phosphorylation, which sustains microglial phagocytic capacity and promotes an anti-inflammatory phenotype (Zhang et al., 2025). Conversely, in the acute phase of intracerebral ICH, HK2 is significantly downregulated at day 1 and day 3 post-ICH, resulting in impaired glycolysis and reduced glucose-6-phosphate production. This metabolic deficit triggers mitochondrial dysfunction, ROS accumulation, and amplified release of pro-inflammatory cytokines (e.g., IL-1β, IL-6, TNF-α), thereby exacerbating neural injury (Li Y. et al., 2024). Notably, a partial recovery of HK2 expression was observed specifically in microglia by day 7, suggesting a time-dependent shift in metabolic reprogramming. To investigate whether this expression pattern of HK2 regulation could be generalized to other hemorrhagic conditions, we employed a SAH model as a complementary approach. Consistent with this temporal dynamic, our early SAH model (day 1) revealed an increase in microglial proportion accompanied by decreased HK2 expression (Supplementary Figures S3A–D), while the ICH model (day 14) showed elevated HK2 levels (Supplementary Figure S3E) in microglia. These contrasting responses across hemorrhagic models highlight a common pattern of HK2 regulation characterized by an initial decline followed by a later rise, with day 7 representing a critical transition point in the metabolic and inflammatory evolution of hemorrhagic stroke. Meanwhile, HSPA5 (also known as GRP78) serves a protective role by mitigating endoplasmic reticulum (ER) stress and inhibiting neuronal ferroptosis. Following ICH, HSPA5 is upregulated as part of the unfolded protein response to counteract ER stress-induced apoptosis, which directly binds to and stabilizes GPX4, a key regulator of ferroptosis, thereby reducing lipid peroxidation and iron-dependent neuronal death (Li J. et al., 2024). Its expression is modulated by various miRNAs, such as miR-181b and miR-378a-5p, forming critical regulatory axes that influence neuronal survival (Wang et al., 2019; Wang et al., 2022).

To further elucidate the dynamic and spatial coordination of these key players, we employed pseudotemporal trajectory analysis as a hypothesis-generating tool. The analysis indicated that Tnf and Hk2 exhibit coordinated expression dynamics along the inflammatory differentiation pathway, while Hspa5 shows an opposing trend. This inverse relationship could suggest potential antagonistic regulation between ER stress response and glycolytic metabolism during microglial activation, possibly representing a metabolic checkpoint that influences inflammatory outcomes. To explain this, we propose a potential mechanism: HSPA5, as a central ER chaperone, likely regulates HK2 and TNF through two main pathways. Firstly, HSPA5 may trigger chaperone-mediated autophagy (CMA) to degrade glucose metabolic enzymes, suppressing glycolysis—this is supported by findings that HSPA5 activation enhances AMPK-driven autophagy, leading to metabolic reprogramming (Cook and Clarke, 2012; Binder and Pedley, 2023). Secondly, HSPA5 could modulate UPR signaling (e.g., via ERN1/JNK) to sustain TNF-α production, as knockdown experiments reduce inflammation in ICH models (Wang et al., 2022). This reinforces HSPA5’s role as a metabolic-inflammatory integrator. However, as pseudotime analysis is inferential and sensitive to parameters, it requires validation with temporal data, motivating our further spatial transcriptomics approach. However, it should be mentioned that pseudotime analysis is inherently inferential and sensitive to parameters such as starting cell selection; it does not equate to biological time and should be interpreted with caution, as evidenced by methodological limitations discussed in Barile et al. (2021) and Zheng et al. (2023). Therefore, these observations are speculative and require validation through complementary temporal data, which prompted our subsequent spatial transcriptomics investigation across multiple timepoints.

Spatial transcriptomic analysis delineated the anatomical distribution of molecular alterations following ICH. Based on the predominant focus of ICH pathophysiology on inflammation and lipid pathways, no significant changes were observed in glucose metabolism-related genes, such as HK2, underscoring those metabolic adaptations. Thus, when we specifically focus on glucose metabolism, our spatial and temporal analysis (from 3 h to 28 days) reveals that HK2 upregulation in perihematomal regions is an early and dynamic response, with expression differences between ipsilateral and contralateral sides beginning to narrow by day 3 and declining rapidly by day 7. This observation can be partially explained by the findings of Askenase et al. (2021), which demonstrated that HK2 expression is elevated in the acute phase (within 4 days post-ICH) in CD14^+^ monocytes/macrophages and neutrophils within the hematoma, but decreases over time as the response transitions to a sub-acute stage. The apparent HK2 elevation in our spatial data may reflect the inherent resolution limits of spatial transcriptomics, where each ‘spot’ captures a mixture of cell types, including infiltrating inflammatory cells that express HK2 under stress, thereby contributing to the perihematomal signal. In this context, our findings may not be contradictory with previous findings, thus, underscoring the conserved nature of metabolic reprogramming towards glycolysis post-ICH. Given this alignment, the perceived discrepancies with other reports likely stem from methodological differences in cellular resolution. While spatial transcriptomics offers valuable spatiotemporal insights, its limitation in distinguishing pure cell populations, which may lead to apparent contradictions due to the inclusion of multiple cell types (e.g., neutrophils or monocytes) that overexpress HK2 under stress. This technical aspect highlights the need for complementary approaches to fully resolve cell-specific responses.

Furthermore, to decipher how these molecular changes influence other cell types, cell-cell communication analysis revealed that among our target genes, TNF signaling undergoes the most substantial reorganization post-ICH. The enhanced microglia-to-neutrophil communication via *Tnf-Tnfrsf1b* pairs suggests a specific mechanism through which activated microglia recruit and prime peripheral immune cells, amplifying the inflammatory cascade. This is supported by the findings of Zhang et al. (Zhang et al., 2024), which identified elevated TNF signaling in neutrophils, with microglia-derived osteopontin interacting with CD44 on monocytes, but also emphasized TNF-TNFRSF1B as a key pathway driven by microglia-monocyte crosstalk in ICH. However, it is important to note that our CellChat analysis is based on transcriptomic data, which provides valuable insights into potential communication pathways but does not directly demonstrate protein-level interactions or causal relationships. While existing evidence supports the functional importance of TNF signaling in ICH pathophysiology - including studies showing *Tnf-Tnfrsf1b* signaling exacerbates neuroinflammation through exosomal pathways (Zhu et al., 2024; Puy et al., 2022; Kawamura et al., 2024) and our own data showing elevated inflammatory cytokines - we acknowledge that these findings remain inferential regarding actual protein-level interactions. Future studies employing targeted approaches such as co-culture experiments, protein interaction assays, or conditional knockout models will be essential to validate the functional significance of the TNF-TNFRSF1B axis in microglia-neutrophil communication. Such investigations could specifically test whether disruption of this pathway attenuates neuroinflammation and improves outcomes in ICH models. On the other hand, the absence of significant changes in *Hspa5* and *Hk2*-associated pathways indicates that while these genes participate in cellular adaptation, they may not drive direct intercellular signaling to the same extent as TNF. This distinction highlights the importance of differentiating intracellular metabolic adaptation from intercellular communication in understanding neuroinflammation.

Our study has several limitations that should be considered when interpreting the findings. First, the integrative multi-omics approach utilizes data from different species (human bulk RNA-seq, mouse single-cell and spatial transcriptomics), technologies, and time points. We mention that our primary goal was conceptual integration and hypothesis generation, not directly quantitative cross-species comparison. Although we applied state-of-the-art batch correction and normalization methods, inherent biological differences between species (e.g., neuroinflammatory responses) and the mismatch in resolution (bulk tissue vs. single-cell vs. spatial transcriptomics) limit the robustness of direct comparisons. We mitigated this by focusing on evolutionarily conserved pathways and core regulatory genes (e.g., the TNF signaling axis) reported across both human and murine studies of neuroinflammation. Second, the temporal dynamics were reconstructed from datasets with non-identical timepoints. While this provided a useful view for analysis, subtle phase differences between human and mouse disease progression may remain. Third, our conclusions are primarily based on transcriptional evidence. Protein-level validation and functional experiments using genetic or pharmacological perturbations are required to confirm the mechanistic roles of the identified genes in our further study. Finally, the use of public datasets, despite rigorous quality control and batch effect correction, may carry unresolved technical variation. Future studies employing uniform platforms, matched time-series, and cross-species validation at the protein and functional levels will be essential to translate these findings.

Future studies should investigate the causal relationships between these metabolic and inflammatory pathways using cell-specific knockout models. The Day 7 transition point identified in our temporal analysis represents a particularly promising target for therapeutic intervention. This time point may correspond to a critical transition from acute inflammation to the initiation of repair and resolution phases post-ICH, a demarcation supported by prior literature on temporal immune dynamics after brain injury. Exploring whether metabolic modulation (e.g., with HK2 inhibitors or ER stress mitigators) can alter microglial polarization states and improve functional outcomes, especially during this potential transition window, could have significant translational potential.

## 5 Conclusion

Our integrated multi-omics analysis demonstrates that glucose metabolism and neuroinflammation are intimately connected in the ICH brain through coordinated changes in key regulators Hk2, Hspa5, and Tnf. These genes exhibit cell-type-specific, spatially organized, and temporally dynamic expression patterns that shape microglial activation states and cellular crosstalk. The identification of a synchronized transition point at Day 7 and the specific microglia-neutrophil signaling axis via TNF provides novel insights into the pathophysiology of ICH and suggests potential targets for future therapeutic strategies aimed at modulating the metabolic-inflammatory interface.

## Data Availability

The datasets presented in this study can be found in online repositories. The names of the repository/repositories and accession number(s) can be found in the article/Supplementary Material.

## References

[B1] Alsbrook D. L. Di Napoli M. Bhatia K. Biller J. Andalib S. Hinduja A. (2023). Neuroinflammation in acute ischemic and hemorrhagic stroke. Curr. Neurology Neuroscience Reports 23 (8), 407–431. 10.1007/s11910-023-01282-2 37395873 PMC10544736

[B2] An S. J. Kim T. J. Yoon B.-W. (2017). Epidemiology, risk factors, and clinical features of intracerebral hemorrhage: an update. J. Stroke 19 (1), 3–10. 10.5853/jos.2016.00864 28178408 PMC5307940

[B3] Askenase M. H. Goods B. A. Beatty H. E. Steinschneider A. F. Velazquez S. E. Osherov A. (2021). Longitudinal transcriptomics define the stages of myeloid activation in the living human brain after intracerebral hemorrhage. Sci. Immunology 6 (56), eabd6279. 10.1126/sciimmunol.abd6279 33891558 PMC8252865

[B4] Bahadar G. A. Shah Z. A. (2021). Intracerebral hemorrhage and diabetes mellitus: blood-brain barrier disruption, pathophysiology and cognitive impairments. CNS and Neurological Disorders-Drug Targets-CNS and Neurological Disord. 20 (4), 312–326. 10.2174/1871527320666210223145112 33622232

[B5] Barile M. Imaz-Rosshandler I. Inzani I. Ghazanfar S. Nichols J. Marioni J. C. (2021). Coordinated changes in gene expression kinetics underlie both mouse and human erythroid maturation. Genome Biology 22 (1), 197. 10.1186/s13059-021-02414-y 34225769 PMC8258993

[B6] Binder M. J. Pedley A. M. (2023). The roles of molecular chaperones in regulating cell metabolism. FEBS Letters 597 (13), 1681–1701. 10.1002/1873-3468.14682 37287189 PMC10984649

[B8] Cook K. L. Clarke R. B. (2012). Heat shock 70 kDa protein 5/glucose-regulated protein 78 “AMP” ing up autophagy. Autophagy 8 (12), 1827–1829. 10.4161/auto.21765 22931685 PMC3541293

[B9] D'Onofrio N. Sardu C. Trotta M. C. Scisciola L. Turriziani F. Ferraraccio F. (2021). Sodium-glucose co-transporter2 expression and inflammatory activity in diabetic atherosclerotic plaques: effects of sodium-glucose co-transporter2 inhibitor treatment. Mol. Metab. 54, 101337. 10.1016/j.molmet.2021.101337 34500107 PMC8473552

[B10] Feigin V. L. Nichols E. Alam T. Bannick M. S. Beghi E. Blake N. (2019). Global, regional, and national burden of neurological disorders, 1990–2016: a systematic analysis for the global burden of disease study 2016. Lancet Neurology 18 (5), 459–480. 10.1016/S1474-4422(18)30499-X 30879893 PMC6459001

[B11] Fujimoto M. Shiba M. Kawakita F. Liu L. Shimojo N. Imanaka-Yoshida K. (2016). Deficiency of tenascin-C and attenuation of blood-brain barrier disruption following experimental subarachnoid hemorrhage in mice. J. Neurosurg. 124 (6), 1693–1702. 10.3171/2015.4.JNS15484 26473781

[B12] Gong Y. Li H. Cui H. Gong Y. (2025). Microglial mechanisms and therapeutic potential in brain injury post-intracerebral hemorrhage. J. Inflamm. Res. 18, 2955–2973. 10.2147/JIR.S498809 40026311 PMC11872102

[B13] Han X. Ren H. Nandi A. Fan X. Koehler R. C. (2021). Analysis of glucose metabolism by 18F-FDG-PET imaging and glucose transporter expression in a mouse model of intracerebral hemorrhage. Sci. Reports 11 (1), 10885. 10.1038/s41598-021-90216-4 34035344 PMC8149426

[B14] Hao Y. Hao S. Andersen-Nissen E. Mauck W. M. Zheng S. Butler A. (2021). Integrated analysis of multimodal single-cell data. Cell 184 (13), 3573–3587. 10.1016/j.cell.2021.04.048 34062119 PMC8238499

[B15] Jia P. Peng Q. Fan X. Zhang Y. Xu H. Li J. (2024). Immune‐mediated disruption of the blood–brain barrier after intracerebral hemorrhage: insights and potential therapeutic targets. CNS Neurosci. and Ther. 30 (7), e14853. 10.1111/cns.14853 39034473 PMC11260770

[B16] Jin S. Plikus M. V. Nie Q. (2025). CellChat for systematic analysis of cell–cell communication from single-cell transcriptomics. Nat. Protocols 20 (1), 180–219. 10.1038/s41596-024-01045-4 39289562

[B17] Kandel S. Paepcke A. Hellerstein J. Heer J. (2011). Wrangler: interactive visual specification of data transformation scripts. Proceedings of the sigchi conference on human factors in computing systems, 3363–3372. 10.1145/1978942.1979444

[B18] Kawamura Y. Johnson C. W. DeLong J. de Lima Camillo L. P. Takahashi M. Beatty H. E. (2024). Single-cell temporal atlas of myeloid cells in the live haemorrhagic brain. bioRxiv. 10.1101/2024.12.24.630187

[B19] Lan X. Han X. Li Q. Yang Q.-W. Wang J. (2017). Modulators of microglial activation and polarization after intracerebral haemorrhage. Nat. Rev. Neurol. 13 (7), 420–433. 10.1038/nrneurol.2017.69 28524175 PMC5575938

[B20] Leek J. T. Johnson W. E. Parker H. S. Jaffe A. E. Storey J. D. (2012). The sva package for removing batch effects and other unwanted variation in high-throughput experiments. Bioinformatics 28 (6), 882–883. 10.1093/bioinformatics/bts034 22257669 PMC3307112

[B21] Lei W. Zhuang H. Huang W. Sun J. (2025). Neuroinflammation and energy metabolism: a dual perspective on ischemic stroke. J. Transl. Med. 23 (1), 413. 10.1186/s12967-025-06440-3 40211331 PMC11983748

[B22] Li X. Wang C.-Y. (2021). From bulk, single-cell to spatial RNA sequencing. Int. Journal Oral Science 13 (1), 36. 10.1038/s41368-021-00146-0 34782601 PMC8593179

[B23] Li Y. Zhou H. He X. Jin L. Zhu Y. Hu L. (2024). Impaired microglial glycolysis promotes inflammatory responses after intracerebral haemorrhage via HK2-dependent mitochondrial dysfunction. J. Adv. Res. 73, 575–591. 10.1016/j.jare.2024.08.016 39142439 PMC12225926

[B24] Li J. J. Lin L. Yu Z. He J. Li Y. Jiang J. (2024). IL-1β-induced mesenchymal stem cell-derived exosomes inhibit neuronal ferroptosis in intracerebral hemorrhage through the HSPA5/GPX4 axis. Brain Res. 1845, 149219. 10.1016/j.brainres.2024.149219 39222871

[B25] Ma X. Nan F. Liang H. Shu P. Fan X. Song X. (2022). Excessive intake of sugar: an accomplice of inflammation. Front. Immunology 13, 988481. 10.3389/fimmu.2022.988481 36119103 PMC9471313

[B26] Morabito S. Reese F. Rahimzadeh N. Miyoshi E. Swarup V. (2023). hdWGCNA identifies co-expression networks in high-dimensional transcriptomics data. Cell Reports Methods 3 (6), 100498. 10.1016/j.crmeth.2023.100498 37426759 PMC10326379

[B27] Namgaladze D. Brüne B. (2023). Rapid glycolytic activation accompanying innate immune responses: mechanisms and function. Front. Immunology 14, 1180488. 10.3389/fimmu.2023.1180488 37153593 PMC10158531

[B28] Ovalı M. A. Perçin Ş. (2024). A disease-based perspective of the relationship between neuroinflammation and impaired glucose metabolism. Ağrı Tıp Fakültesi Derg. 2 (3), 132–136. 10.61845/agrimedical.1527141

[B29] Piwecka M. Rajewsky N. Rybak-Wolf A. (2023). Single-cell and spatial transcriptomics: deciphering brain complexity in health and disease. Nat. Rev. Neurol. 19 (6), 346–362. 10.1038/s41582-023-00809-y 37198436 PMC10191412

[B30] Puy L. Perbet R. Figeac M. Duchêne B. Deramecourt V. Cordonnier C. (2022). Brain peri-hematomal area, a strategic interface for blood clearance: a human neuropathological and transcriptomic study. Stroke 53 (6), 2026–2035. 10.1161/STROKEAHA.121.037751 35465695

[B31] Ritchie M. E. Phipson B. Wu D. Hu Y. Law C. W. Shi W. (2015). Limma powers differential expression analyses for RNA-sequencing and microarray studies. Nucleic Acids Research 43 (7), e47–e. 10.1093/nar/gkv007 25605792 PMC4402510

[B32] Rosell A. Vilalta A. García-Berrocoso T. Fernandez-Cadenas I. Domingues-Montanari S. Cuadrado E. (2011). Brain perihematoma genomic profile following spontaneous human intracerebral hemorrhage. PloS One 6 (2), e16750. 10.1371/journal.pone.0016750 21311749 PMC3032742

[B33] Seiffge D. J. Fandler-Höfler S. Du Y. Goeldlin M. B. Jolink W. M. Klijn C. J. (2024). Intracerebral haemorrhage—mechanisms, diagnosis and prospects for treatment and prevention. Nat. Rev. Neurol. 20 (12), 708–723. 10.1038/s41582-024-01035-w 39548285

[B34] Shao X. Liao J. Lu X. Xue R. Ai N. Fan X. (2020). scCATCH: automatic annotation on cell types of clusters from single-cell RNA sequencing data. Iscience 23 (3), 100882. 10.1016/j.isci.2020.100882 32062421 PMC7031312

[B35] Shi X. Luo L. Wang J. Shen H. Li Y. Mamtilahun M. (2021). Stroke subtype-dependent synapse elimination by reactive gliosis in mice. Nat. Communications 12 (1), 6943. 10.1038/s41467-021-27248-x 34836962 PMC8626497

[B36] Thieren L. Zanker H. S. Droux J. Dalvi U. Wyss M. T. Waag R. (2025). Astrocytic GLUT1 deletion in adult mice enhances glucose metabolism and resilience to stroke. Nat. Commun. 16 (1), 4190. 10.1038/s41467-025-59400-2 40328784 PMC12056070

[B37] Thind A. S. Monga I. Thakur P. K. Kumari P. Dindhoria K. Krzak M. (2021). Demystifying emerging bulk RNA-Seq applications: the application and utility of bioinformatic methodology. Briefings Bioinformatics 22 (6), bbab259. 10.1093/bib/bbab259 34329375

[B38] Tonyan Z. N. Nasykhova Y. A. Danilova M. M. Barbitoff Y. A. Changalidi A. I. Mikhailova A. A. (2022). Overview of transcriptomic research on type 2 diabetes: challenges and perspectives. Genes 13 (7), 1176. 10.3390/genes13071176 35885959 PMC9319211

[B7] Trapnell C. Cacchiarelli D. Grimsby J. Pokharel P. Li S. Morse M. (2014). The dynamics and regulators of cell fate decisions are revealed by pseudotemporal ordering of single cells. Nat. Biotechnol. 32, 381–386. 10.1038/nbt.2859 24658644 PMC4122333

[B39] Tschoe C. Bushnell C. D. Duncan P. W. Alexander-Miller M. A. Wolfe S. Q. (2020). Neuroinflammation after intracerebral hemorrhage and potential therapeutic targets. J. Stroke 22 (1), 29–46. 10.5853/jos.2019.02236 32027790 PMC7005353

[B40] Vallejos C. A. Risso D. Scialdone A. Dudoit S. Marioni J. C. (2017). Normalizing single-cell RNA sequencing data: challenges and opportunities. Nat. Methods 14 (6), 565–571. 10.1038/nmeth.4292 28504683 PMC5549838

[B41] Wang Z. Fang L. Shi H. Yang Z. (2019). miR-181b regulates ER stress induced neuron death through targeting heat shock protein A5 following intracerebral haemorrhage. Immunol. Lett. 206, 1–10. 10.1016/j.imlet.2018.11.014 30503822

[B42] Wang B. Zhao X. Xiao L. Chen Y. (2022). FoxO1 silencing facilitates neurological function recovery in intracerebral hemorrhage mice *via* the lncRNA GAS5/miR-378a-5p/Hspa5 axis. J. Stroke Cerebrovasc. Dis. 31 (7), 106443. 10.1016/j.jstrokecerebrovasdis.2022.106443 35487009

[B43] Wu C.-H. Zhou X. Chen M. (2025). Exploring and mitigating shortcomings in single-cell differential expression analysis with a new statistical paradigm. Genome Biol. 26 (1), 58. 10.1186/s13059-025-03525-6 40098192 PMC11912664

[B44] Xiang R. Wang J. Chen Z. Tao J. Peng Q. Ding R. (2025). Spatiotemporal transcriptomic maps of mouse intracerebral hemorrhage at single-cell resolution. Neuron 113, 2102–2122.e7. 10.1016/j.neuron.2025.04.026 40412375

[B45] Yao L. Yi J. Cheng L. Wang R. Wen J. Liang J. (2025). Novel association between E3 ubiquitin ligase MARCH8 and glucose transporter Glut1 in intracerebral hemorrhage. Int. Immunopharmacol. 158, 114798. 10.1016/j.intimp.2025.114798 40373593

[B46] Yin H. Duo H. Li S. Qin D. Xie L. Xiao Y. (2024). Unlocking biological insights from differentially expressed genes: concepts, methods, and future perspectives. J. Adv. Res. 76, 135–157. 10.1016/j.jare.2024.12.004 39647635 PMC12793742

[B47] Yu G. Wang L.-G. Han Y. He Q.-Y. (2012). clusterProfiler: an R package for comparing biological themes among gene clusters. Omics A Journal Integrative Biology 16 (5), 284–287. 10.1089/omi.2011.0118 22455463 PMC3339379

[B48] Zhang X. Lan Y. Xu J. Quan F. Zhao E. Deng C. (2019). CellMarker: a manually curated resource of cell markers in human and mouse. Nucleic Acids Research 47 (D1), D721–D728. 10.1093/nar/gky900 30289549 PMC6323899

[B49] Zhang P. Gao C. Guo Q. Yang D. Zhang G. Lu H. (2024). Single-cell RNA sequencing reveals the evolution of the immune landscape during perihematomal edema progression after intracerebral hemorrhage. J. Neuroinflammation 21 (1), 140. 10.1186/s12974-024-03113-8 38807233 PMC11131315

[B50] Zhang W. Wang X. Zhang B. Yi M. Lu Y. Wang S. (2025). Single-cell RNA-seq revealed the immune microenvironment reprogramming by dexmedetomidine treatment in ischemic stroke. Mol. Neurobiol. 62, 1–18. 10.1007/s12035-025-05237-1 40751033 PMC12559068

[B51] Zheng H. Vijg J. Fard A. T. Mar J. C. (2023). Measuring cell-to-cell expression variability in single-cell RNA-sequencing data: a comparative analysis and applications to B cell aging. Genome Biology 24 (1), 238. 10.1186/s13059-023-03036-2 37864221 PMC10588274

[B52] Zhou Y. Wang Y. Wang J. Stetler R. A. Yang Q.-W. (2014). Inflammation in intracerebral hemorrhage: from mechanisms to clinical translation. Prog. Neurobiology 115, 25–44. 10.1016/j.pneurobio.2013.11.003 24291544

[B53] Zhu X. Xu Z. Liu Y. Yang J. Bai L. Li X. (2024). Unveiling microglia heterogeneity in intracerebral hemorrhage. Neuroscience 555, 167–177. 10.1016/j.neuroscience.2024.07.039 39067680

