# Supplementary material for: Integrative transcriptomic analysis reveals microglial metabolic-inflammatory crosstalk of HK2–HSPA5–TNF axis after intracerebral hemorrhage
Source: Front Bioinform. 2026 Jan 12;5:1740715. doi: 10.3389/fbinf.2025.1740715 (PMC12833071; doi:10.3389/fbinf.2025.1740715)
Supplement: Supplementary file 2 [file DataSheet2.docx]

**Supplementary Figure 1**


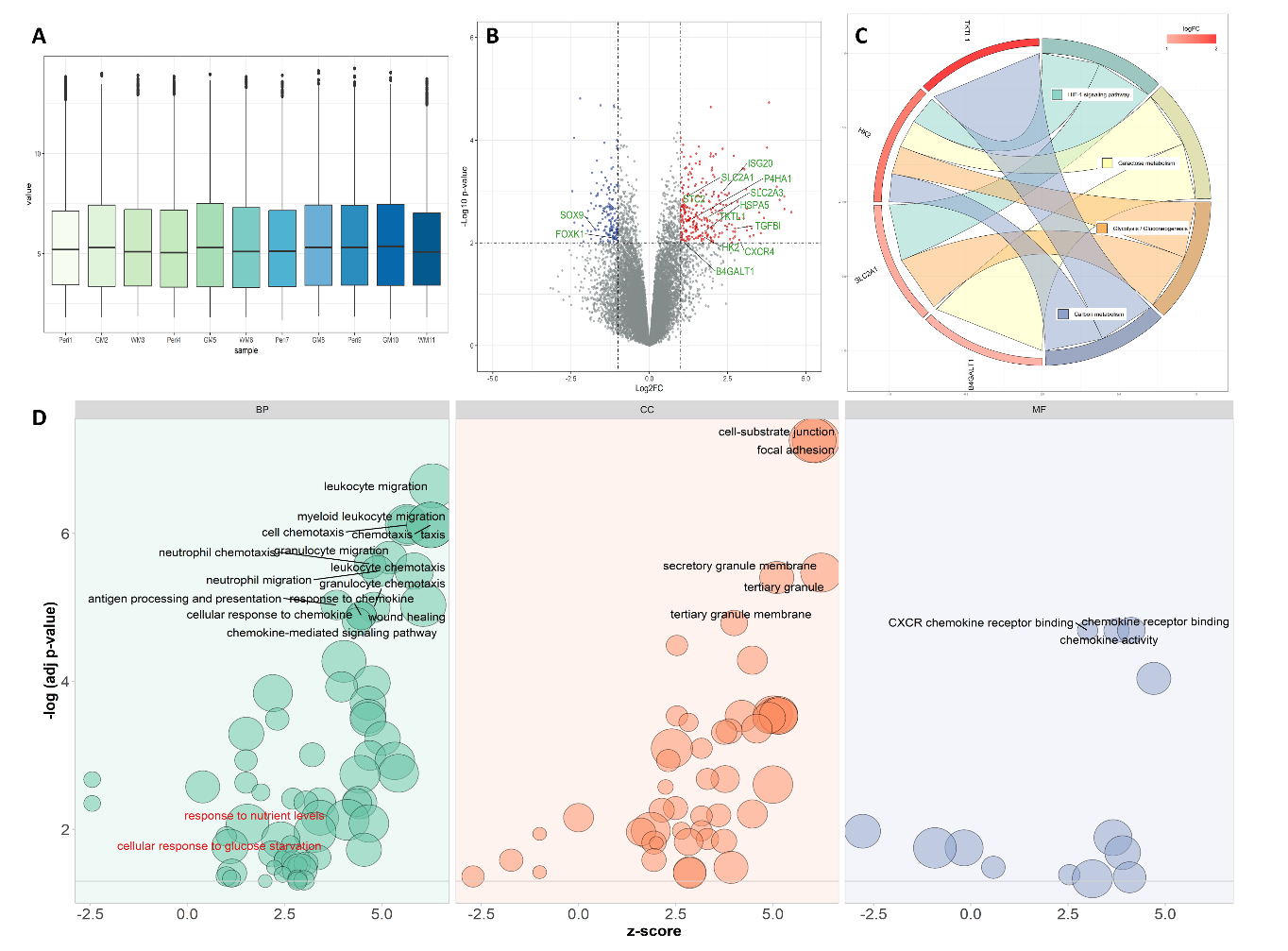


**Fig. S1.** Transcriptomic alterations after ICH.​​ **(A)​​** Boxplots shows expression distribution across samples. ​​**(B)​​** Volcano plot of differentially expressed genes with glucose metabolism related genes (e.g., HK2, HSPA5) are labeled. **​​​(C)​​** Chord diagram illustrating associations between selected genes and KEGG functional pathways. **​​(D)​​** GO enrichment analysis across Biological Process (BP), Cellular Component (CC), and Molecular Function (MF) categories. Bubble size represents gene count. A positive z-score indicates term significantly associated with upregulation, whereas a negative z-score indicates association with downregulation.​​ Key glucose metabolism-related terms are highlighted.

**Supplementary Figure 2**


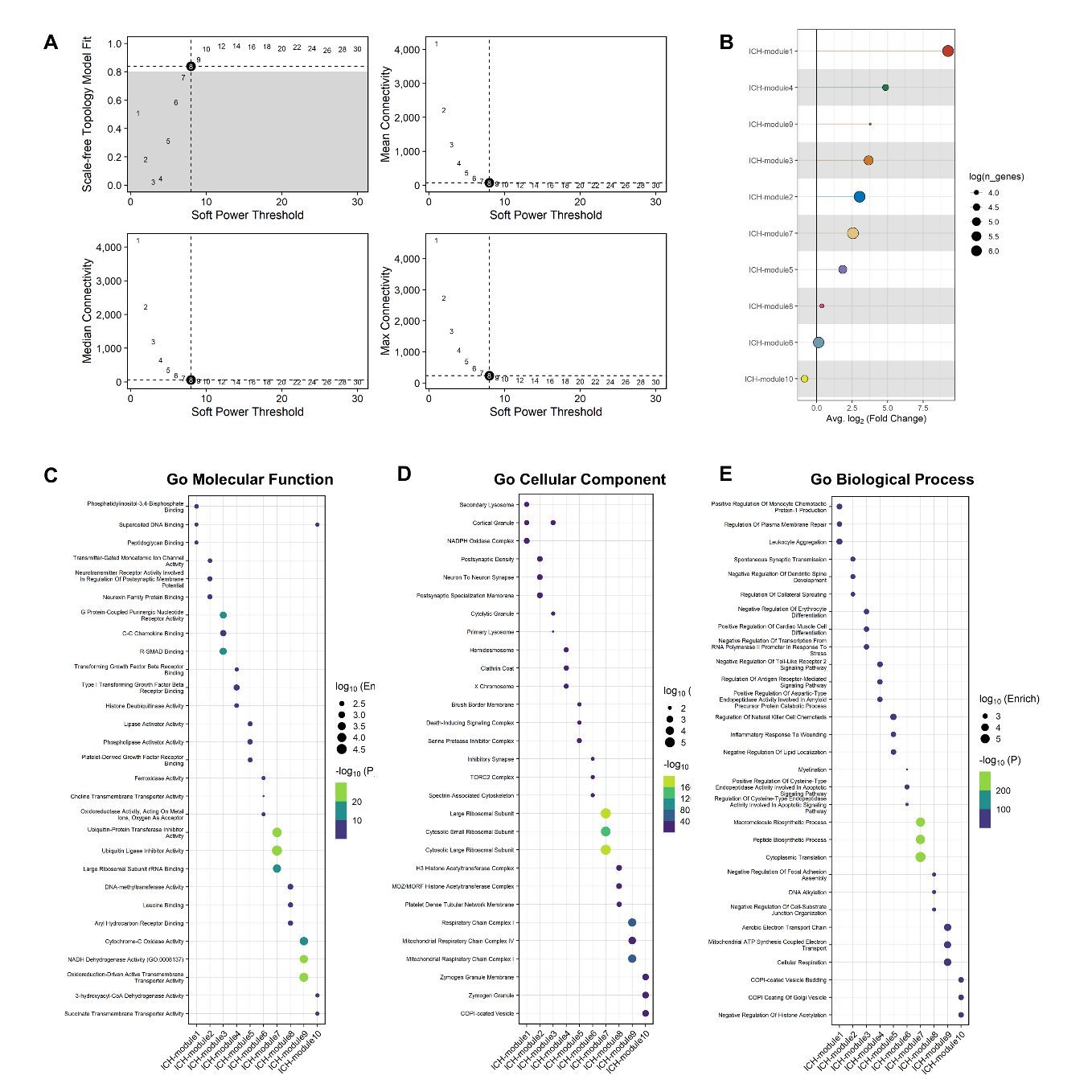


Fig. S2. Analyses of WGCNA network construction and functional enrichment. **(A)​​** Soft thresholding analysis for network construction. (B)​​ Lollipop plot of module eigengene expression versus average log₂(fold change) between ICH and control conditions. Each point represents a module, colored by module identity, with size corresponding to the number of genes in each module. **(C-E)​​** GO enrichment analysis for modules. Point size represents enrichment fold change, and color indicates -log₁₀ (P value).

**Supplementary Figure 3**


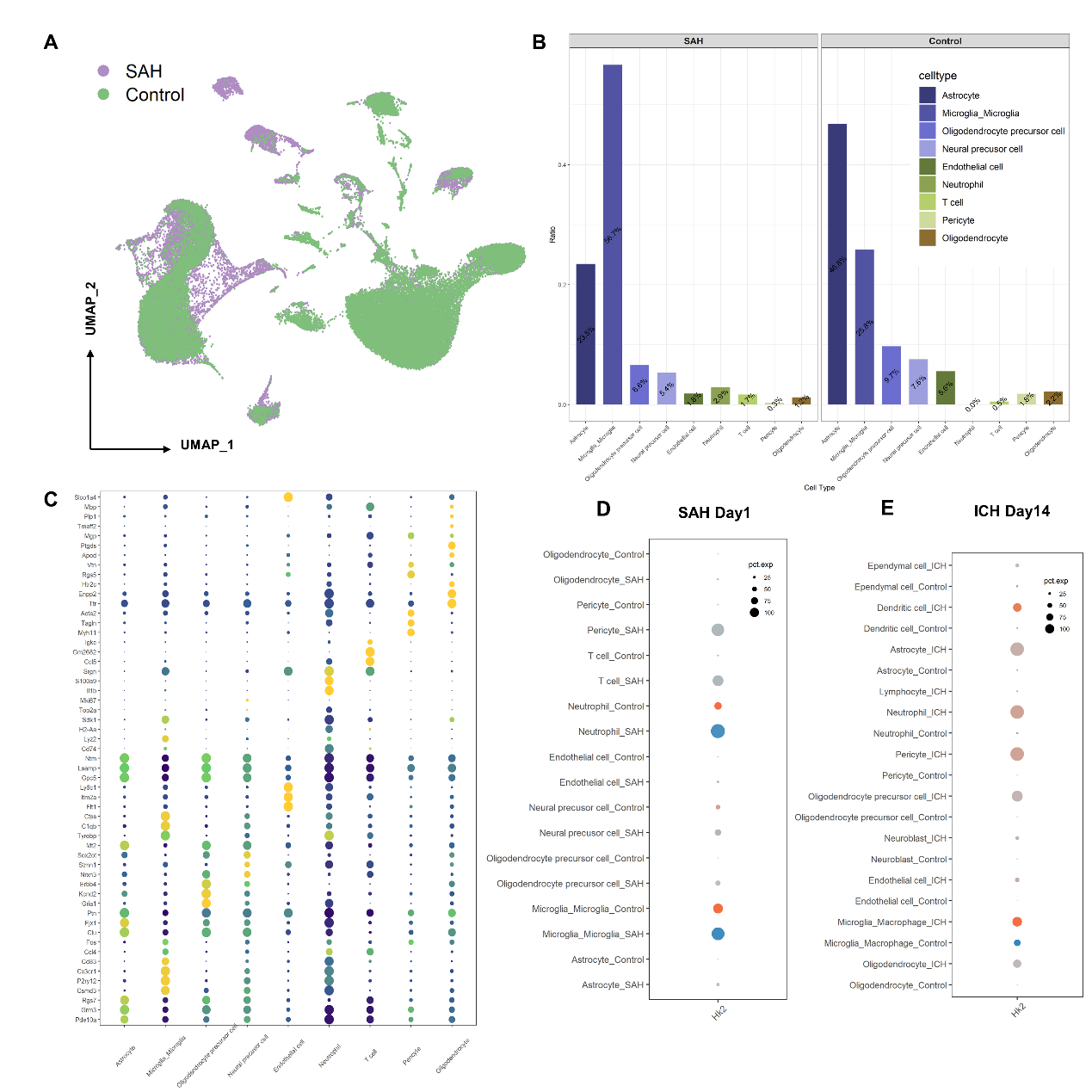


Fig. S3. The scRNA profiling of cell composition and gene expression changes in SAH and ICH models.​​

​​**(A)​​** UMAP projection showing the distribution of cells from SAH (purple) and sham-control (green) groups. **(B)​​** Bar plot comparing the proportional abundance of major cell types between SAH and control conditions. **​​(C)**​​ Dot plots displays gene markers of cell clusters. **(D)** Dot plots illustrating the cell HK2 expression levels across cell types 1 day after SAH. **(E)** Dot plots illustrating the cell HK2 expression levels across cell types 14 days after ICH.
